# Remote Lifestyle Coaching Plus a Connected Glucose Meter with Certified Diabetes Educator Support Improves Glucose and Weight Loss for People with Type 2 Diabetes

**DOI:** 10.1155/2018/3961730

**Published:** 2018-05-16

**Authors:** Jennifer B. Bollyky, Dena Bravata, Jason Yang, Mark Williamson, Jennifer Schneider

**Affiliations:** ^1^Livongo Health, Mountain View, CA, USA; ^2^Stanford University, Hospital & Clinics, Stanford, CA, USA; ^3^Center for Primary Care and Outcomes Research, Stanford University, Stanford, CA, USA; ^4^Biomedical Informatics, Stanford University, Stanford, CA, USA; ^5^Restore Health, Palo Alto, CA, USA

## Abstract

**Background:**

Connected health devices with lifestyle coaching can provide real-time support for people with type 2 diabetes (T2D). However, the intensity of lifestyle coaching needed to achieve outcomes is unknown.

**Methods:**

Livongo provides connected, two-way messaging glucose meters, unlimited blood glucose (BG) test strips, and access to certified diabetes educators. We evaluated the incremental effects of adding lifestyle coaching on BG, estimated HbA1c, and weight. We randomized 330 eligible adults (T2D, HbA1c > 7.5%, BMI ≥ 25) to receive no further intervention (*n* = 75), a connected scale (*n* = 115), scale plus lightweight coaching (*n* = 73), or scale plus intense coaching (*n* = 67) for 12 weeks. We evaluated the change in outcomes using ANOVA.

**Results:**

Livongo participation alone resulted in improved BG control (mean HbA1c declined: 8.5% to 7.5%, *p* = 0.01). Mean weight loss and additional BG decreases were higher in the intensive compared with the lightweight coaching and scale-only groups (weight change (lb): −6.4, −4.1, and −1.1, resp., *p* = 0.01; BG change (mg/dL): −19.4, −11.3, and −2.9, resp., *p* = 0.02). The estimated 12-week program costs were 5.5 times more for intensive than lightweight coaching.

**Conclusion:**

Livongo participation significantly improves BG control in people with T2D. Additional lifestyle coaching may be a cost-effective intervention to achieve further glucose control and weight loss.

## 1. Background

In the USA, 1 in 11 people have diabetes, costing the healthcare system over $150 billion annually [1–3]. For these patients, maintaining blood glucose in the normal range (80–180 mg/dL) [4] is a critical part of reducing emergency department visits, hospitalizations, renal failure, and other costly complications of diabetes [5, 6]. Wirelessly connected glucose meters combined with certified diabetes educator (CDE) support for managing daily fluctuations in blood glucose (e.g., Livongo for diabetes program) have demonstrated sustained improvement in glucose control by reducing HbA1c by 0.9% for up to 12 months [7]. However, not all patients achieve goal glucose control with this type of support alone and many patients with T2D find weight loss in the setting of insulin and other glucose-lowering medications to be difficult.

Lifestyle coaching through the National Diabetes Prevention Program is increasingly recommended to help patients with prediabetes and/or metabolic syndrome change their diets, increase physical activity, improve coping skills, and adopt other key behaviors associated with metabolic improvements and long-term outcomes [8, 9]. To our knowledge, there has been no published evaluation of effects of lifestyle coaching for people diagnosed with type 2 diabetes in the setting of a connected glucose meter and CDE support.

Large healthcare provider systems such as Sutter Health and the Veterans Health Administration (VHA) have made enormous investments in coaching and other educational programs for patients with diabetes. The VHA, for example, has grown its telehealth and remote patient monitoring from an initial program of 2000 patients in 2003 to over 150,000 in 2012 [10]. Patients with diabetes represent the largest population of veterans served through these programs, accounting for 48% of telehealth/remote monitoring visits per year. The annual cost to deploy these programs is $1600 per patient per year—which represents a considerable investment but one that is associated with a 20% reduction in hospital admissions related to diabetes [10] and is much lower than historical alternatives (e.g., over $13,000 per patient per year for traditional home-based care).

Increasingly, self-insured employers are investing in similar programs for their employees with diabetes [11]. Highly customized programs that are able to personalize outreach to target populations, match the intensity of support to the need of the employees, and optimize individuals' blood glucose readings and overall metabolic control are more likely to be cost-effective than with intensive programs deployed to a broad population.

We evaluated the effects of adding lifestyle modification coaching for a population of people with type 2 diabetes enrolled in a program supported by connected glucose meters and CDEs who had not yet achieved their glycemic control and weight goals. We hypothesized that intensive lifestyle coaching would be associated with greater improvements in glycemic control and weight loss than either lightweight coaching, scale-only or no further intervention. Secondarily, we investigated which patient characteristics are associated with the greatest engagement with the lifestyle coaching program.

## 2. Methods

### 2.1. Interventions

#### 2.1.1. Livongo Diabetes Program

The Livongo for the diabetes program offers participants (1) a connected two-way messaging device that measures blood glucose, centrally stores the glucose data and other contextual data (e.g., time of day, relationship to a meal, insulin dose, and carbohydrates eaten), and delivers relevant messages back to the patient; (2) unlimited glucose test strips; and (3) access to a team of CDEs who are available to answer patient questions, help with goal setting, and provide immediate support in the setting of extreme glucose excursions. The Livongo program was provided to participants by their employer or health plan at no cost to the participant.

Algorithmic-driven messages are sent through the Livongo meter in response to each BG reading. For example, if the BG value is <50 mg/dL, the message on the BG meter could read “Your reading is very low, drink 4 oz of juice or take 4 glucose tabs and check BG again in 15 minutes”. If the BG value is >400 mg/dL, the message could read “Drink a glass of water, take medication as prescribed and check BG again in 30 minutes”. When a patient has a BG value of >400 mg/dL or <50 mg/dL or when the BG value is outside the specific threshold the patient sets, an alert is sent to a Livongo CDE who then contacts the patient to ensure the patient's safety and discuss an appropriate action based on the BG value. Twenty-seven percent of Livongo patients have been contacted at least once by a CDE on the basis of an alert BG value. Livongo patients can also schedule 1 : 1 telephonic sessions with CDEs for goal setting, personalized feedback, and diabetes education.

#### 2.1.2. Restore Health Lifestyle Modification Program

The two 12-week lifestyle coaching interventions evaluated in this study were provided by Restore Health (Palo Alto, California) between May and September 2016. Lifestyle modification focused on the four key factors that drive insulin resistance: nutrition, exercise, sleep, and stress. While these topics are addressed according to the AADE7 Self-Care Behavior guidelines followed by Livongo CDEs, the Restore Health nutritional approach recommends a specific diet that is lower in carbohydrates, especially refined carbohydrates and sugar, moderate in protein, and unrestricted in healthy fats. There is no calorie restriction or macronutrient counting involved. Meals were photographed by participants and rated by Restore Health coaches using emojis they texted suggestions to provide real-time feedback to participants. Participants in the intensive coaching arm received a 60-minute onboarding call covering numerous lifestyle-related topics and goal setting and customized lessons as well as daily personalized text messages, personalized meal ratings, and activity recommendations. Participants in the lightweight coaching arm received a 20-minute onboarding call, standardized lessons and templated text message support, meal ratings, and activity recommendations.

#### 2.1.3. Connected Scale

We provided a connected scale (BodyTrace Inc.) to participants in the three intervention arms of this study but not the control/Livongo-only group. Participants were allowed to keep the scale after the pilot program finished. For participants in the control/Livongo-only group, preintervention height and weight were obtained by self-report but no weight values were obtained at the end of the intervention period.

### 2.2. Study Design

1936 adults (age ≥ 21 years) with type 2 diabetes who had been in the Livongo program (between October 1, 2014, and February 26, 2016) for at least 80 days, were overweight (BMI ≥ 25) and had not yet achieved their target glucose control (i.e., estimated HbA1c < 7.0%) were invited to participate in the study. Of these, 454 subjects agreed to participate and were randomized to one of the four intervention groups. 330 subjects provided sufficient data for analysis: (1) Livongo program with no additional support (control group, *N* = 75), (2) Livongo program plus a connected scale (*N* = 115), (3) Livongo program plus a connected scale and 12 weeks of lightweight lifestyle coaching (*N* = 73), or (4) Livongo program plus a connected scale and 12 weeks of intensive lifestyle coaching (*N* = 67).

We collected demographic data (e.g., gender and age), weight, height, and diabetes information (e.g., diabetes type, insulin use, and most recent HbA1c) from participants. Blood glucose data were collected directly from members through their connected Livongo blood glucose meter. Weight data were collected through a connected scale for all participants who were given a scale and by self-report for participants in the control arm. All participants used the same version of the glucose meter, and no significant device or algorithmic changes were made during the study period. No specific guidelines about blood glucose testing frequency were given to participants; rather, they were instructed to follow their healthcare providers' advice. This protocol (LDR-2016) received IRB approval from Aspire Independent Review Board (Santee, CA).

### 2.3. Outcome Measures and Statistical Methods

We performed ANOVA to assess the extent of group assignment on the key outcomes of interest. We performed logistic regression to evaluate the characteristics of individuals with significant improvements in mean blood glucose, estimated HbA1c, and weight. We conducted all statistical analyses in R [12].

#### 2.3.1. Estimated HbA1c

We used a linear model published from the ADAG study [13] to convert the mean blood glucose over 90 days to estimate HbA1c for participants prior to and after the intervention period. The mean blood glucose values prior to the intervention were calculated by taking the average of the blood glucose levels 30 days prior to the participant's enrollment in the study. Similarly, the blood glucose levels from the last 30 days of the study were used as the post-intervention mean blood glucose levels. When there was more than 1 blood glucose value on a given day, the average was taken to be the blood glucose for that day.

#### 2.3.2. Weight Data from Connected Scales

9182 weight values were received from 230 participants. Participants who transmitted fewer than two weight values over the intervention period were excluded from analyses (number of participants removed = 8). We took several steps to assure that data received from connected scales were from the study participant and not another household member. First, transmitted weights at program initiation were compared to self-reported weights to determine a reference starting weight. Second, transmitted weight values more than 20% off of the participant's median weight were excluded (number of weights removed = 436, number of participants removed = 1). Third, if a weight change greater than 3% occurred over one day, transmitted weight values were excluded (number of weights removed = 184). Thus, a total of 8554 weight values from 221 participants were used in the analyses. Sample weight values transmitted from a single scale are shown in Supplementary Figure 1.

#### 2.3.3. Engagement

Program engagement in the lifestyle coaching intervention groups was assessed subjectively by coaches (i.e., “engaged” or “not engaged”) based on program participation, frequency of meal ratings, logged activity level, and sleep. The engagement was quantitatively assessed by the frequency of conversations recorded between coaches and participants.

Diabetes empowerment was evaluated using the Diabetes Empowerment Scale (DES), an eight-item measure of the self-efficacy of people with diabetes, prior to Livongo enrollment and during month 6 of the program [14]. A higher value indicates more positive feelings of empowerment regarding diabetes management.

## 3. Results

Table 1 presents the demographic data for the 330 participants. Baseline characteristics among groups were similar except that the participants randomized to the lightweight coaching intervention were significantly less likely to be on insulin than control participants (*p* = 0.007).

**Figure 1:**
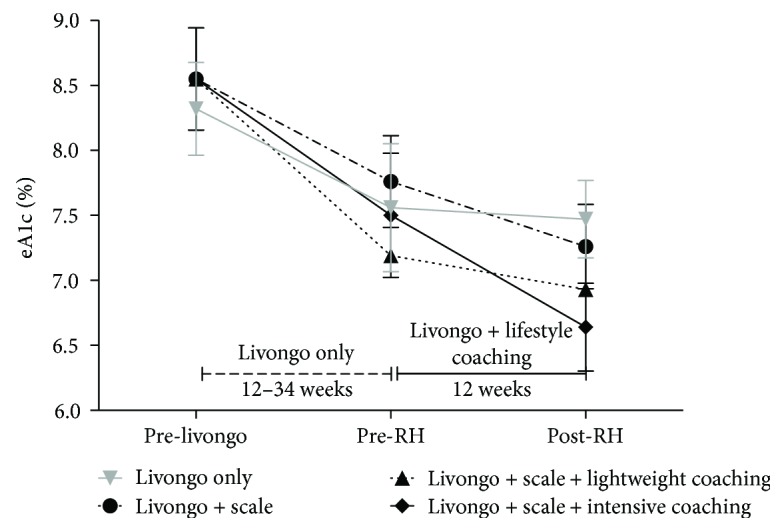
Estimated HbA1c (eA1c) change over time by the intervention group.

Figure 1 presents the change in glucose control for all participants. Livongo participation resulted in significantly improved BG control with a mean estimated HbA1c decrease from 8.5% to 7.5% (*p* = 0.01) across all groups prior to the Restore Health lifestyle modification intervention.  Table 2 presents the change in the key weight and glucose control outcomes for participants in the control and intervention arms. Both coaching arms had statistically significant improvements in weight and additional mean blood glucose compared with the Livongo only and the Livongo plus scale groups. The mean weight loss was greatest in the intensive coaching group (−6.4 ± 9.7 lb) compared to the lightweight coaching (−4.1 ± 9.4 lb) and the Livongo plus scale-only groups (−1.1 ± 13.7 lb) (*p* = 0.005 for intensive coaching compared to scale-only). The additional mean improvement in blood glucose was highest in the intensive coaching group as well (−19.4 ± 34.6 mg/dL) compared to the lightweight coaching (−11.31 ± 34.7 mg/dL) and the Livongo plus scale-only (−2.8 ± 46.9 mg/dL) groups (*p* = 0.02 for intensive coaching compared to scale-only). Interestingly, there were no significant differences between the lightweight and intensive lifestyle coaching groups on any of the outcomes of interest; all statistically significant differences were between the coaching arms and the control group.

We evaluated the characteristics of those participants who had statistically significant improvements in mean blood glucose at the end of the intervention. High pre-intervention mean blood glucose was significantly associated with improved blood glucose control at the end of the intervention (177 ± 52 mg/dL versus 139 ± 26 mg/dL, *p* = 0.001). Age, gender, race/ethnicity, and insulin use were not associated with improvements in mean blood glucose.

**Figure 2:**
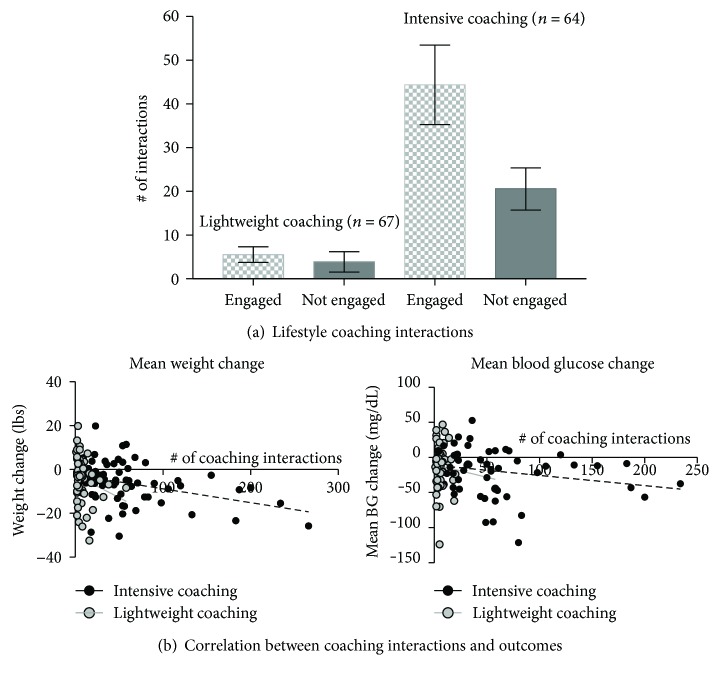
(a) Frequency of lifestyle coaching interactions correlated by engagement level and intervention arm. (b) Engagement with a Restore Health coach is correlated with weight loss and BG change.

Within each of the coaching groups, participants deemed subjectively “engaged” by Restore Health coaches had a significantly higher number of coach interactions with their Restore Health coaches and the number of coach interactions was associated with positive outcomes as shown in Figure 2. The mean number of coach interactions over the 12-week program for the intense coaching group was 44 (range 5–136) compared to 8 (range 1–32) for the lightweight coaching group. The number of coach interactions was strongly correlated with weight loss in the intensive coaching group (Pearson's *r* = −0.36, *p* = 0.003) and to a lesser degree in the lightweight group (Pearson's r = −0.22, *p* = 0.11), but it was not significantly correlated with a change in BG.

**Figure 3:**
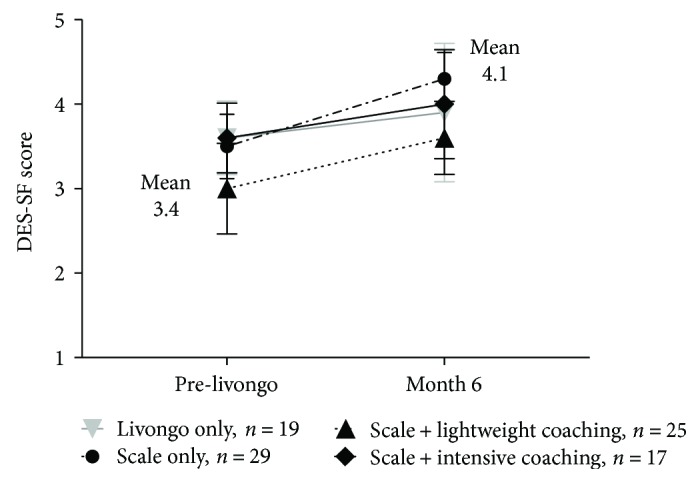
Diabetes Empowerment Scale (DES-SF). The DES-SF asks respondents on a 5-point scale how much they agree with eight statements related how they feel they are coping with diabetes (strongly disagree = 1, strongly agree = 5). Higher scores are associated with greater empowerment. Individual intervention groups did not vary significantly from each other, however the overall mean DES-SF score at registration was significantly lower than at that at 6 months after registration (0.7 difference, *p* = 0.03).

Diabetes empowerment as measured by the DES-SF instrument improved for all participants from a mean value of 3.4 at baseline to 4.1 at 6 months. Areas of the biggest improvement were related to participants' motivation for caring for their diabetes and positive coping strategies. No statistically significant differences between groups were noted as shown in Figure 3.

The incremental cost differences between the two coaching intervention arms were assumed to be related primarily to the coaching time as the fixed costs for the two programs were otherwise the same (i.e., lesson creation, platform for tracking progress towards goals, and connected scale). We further assumed that coaching costs were a function of estimated coach time per text (3 minutes), cost of coaching time ($0.60 per minute) based on $43,500 salary per year [15], 15% fringe benefits, and 70% work hours (40 per week) spent coaching. The mean number of text messages exchanged between coaches and participants was 44 for the intensive coaching group, 8 for the lightweight coaching group, and 0 for the scale-only group.

Based on these assumptions, the 12-week incremental costs above the Livongo program were $92 for the scale-only group, $120 for the scale plus lightweight coaching, and $240 for the scale plus intensive coaching. This translates into $84, $29, and $38 per pound lost; $31.72, $10.62, and $12.37 per mean change in 1 mg/dL of BG; and $230, $300, and $329 per 1% decrease in estimated HbA1c for the scale-only, the lightweight, and the intensive coaching intervention groups, respectively. Though the overall intervention effect size was greatest in the intensive coaching arm, lightweight coaching was more cost-effective for weight loss and change in mean BG.

Based on literature estimates of cost savings attributable to HbA1c reduction ranging from $113 to $179 per member per month [16], lifestyle coaching may be a cost-effective adjunctive therapy for reaching target glucose control for selected participants.

## 4. Discussion

This study has four key findings: first, participation in the Livongo Diabetes program resulted in significant improvement in blood glucose control. For many participants, this program alone is sufficient to achieve desired blood glucose control [7].

Second, among those participants who did not achieve their goal HbA1c through the Livongo program alone, weight loss and glucose control were only significantly greater in the intensive lifestyle coaching group compared to the other groups. The intensive program resulted in better, but nonstatistically significant, outcomes than lightweight coaching. Future studies should be directed to understanding the key components of the lifestyle coaching program most associated with the outcomes of interest to further reduce costs while maintaining effectiveness.

Third, those participants with the greatest improvements in glucose control at the end of the intervention had higher preintervention mean blood glucose levels. This key insight can enable risk stratification of patients entering a program like Livongo to identify the population who might require adjunctive lifestyle counseling to achieve their glucose control goals. By selectively offering lifestyle coaching to this group, the more expensive intervention can be applied to those participants likely to receive the greatest benefit from it.

Finally, all groups experienced an improvement in their sense of empowerment and well-being about managing their diabetes. We attribute this to the personalized digital and telephonic diabetes advice available to all participants through the Livongo-certified diabetes educators.

The limitations of this study include that glucose control at the end of the intervention was calculated from daily blood glucose values rather than measured by laboratory HbA1c. Notably, estimated HbA1c values have been previously demonstrated to be highly correlated with directly measured HbA1c values [13]. Additionally, the population that had the greatest improvement in glucose control may have received additional outside interventions by their primary care and other health care providers that could have contributed to this finding. We did not find significant differences in the frequency of interaction with Livongo coaches either through alerts or scheduled coaching between the groups, but other unmeasured interventions may have occurred.

Future research should be directed at understanding the key elements of the intensive lifestyle coaching program most associated with improvements in weight and glucose control so that those elements can be provided in the most cost-effective means possible to the T2D population most likely to benefit from them.

## Figures and Tables

**Table 1:** Descriptive characteristics of the study populations.

	All participants	Livongo only	Livongo + scale	Livongo + scale + lightweight coaching	Livongo + scale + intensive coaching	ANOVA *p* value
*N*	330	75	115	73	67	
Gender, female	184 (55.8%)	45 (60%)	57 (49.6%)	38 (52.8%)	44 (65.7%)	0.14
Mean age (years)	50.3 ± 9.6	52.8 ± 11.2	49.8 ± 9.5	51.3 ± 9.8	49.9 ± 9.74	0.19
Race						
White/caucasian	212 (64.2%)	45 (60%)	73 (63.5%)	50 (68.5%)	44 (65.7%)	0.74
Hispanic/latino/mexican	3 (0.9%)	0 (0%)	2 (1.7%)	0 (0%)	1 (1.5%)	0.48
Black/african american	36 (10.9%)	9 (12%)	11 (9.6%)	10 (13.7%)	6 (9%)	0.76
Unknown race	39 (11.8%)	14 (18.7%)	18 (15.7%)	2 (2.7%)	5 (7.5%)	0.51
Insulin use						
No insulin use	189 (57.3%)	36 (48%)	57 (49.6%)	52 (71.2%)^∗^	44 (65.7%)	**0.007**
Once/day insulin use	58 (17.6%)	14 (18.7%)	21 (18.3%)	10 (13.7%)	13 (19.4%)	0.80
More than once/day insulin use	71 (21.5%)	25 (33.3%)	27 (23.5%)	11 (15.1%)^∗^	8 (11.9%)	**0.008**
Mean number of days in Livongo program at start of intervention	229 ± 130	217 ± 85	228 ± 143	233 ± 138	243 ± 145	0.71

^∗^There are no statistically significant differences in these baseline characteristics among groups except that the participants randomized to the lightweight intervention were significantly less likely to be on insulin than control participants.

**Table 2:** Change in weight and glucose control with intervention.

Outcomes	All participants	Livongo only	Livongo + scale	Livongo + scale + lightweight RH coaching	Livongo + scale + intensive RH coaching	ANOVA *p* value
Weight (lb)						
*N*	221	NA	90	67	64	
Weight, preintervention	236 ± 52	NA	224 ± 52	246 ± 49	244 ± 55	**0.02**
Weight, postintervention	233 ± 51	NA	223 ± 50	242 ± 49	238 ± 53	**0.06**
Weight change	−3.5 ± 11.6	NA	−1.1 ± 13.7	−4.1 ± 9.4	−6.4 ± 9.7	**0.02**

BG checks per day						
*N*	324	75	113	71	65	
BG count, preintervention^∗^	1.05 ± 1	1.2 ± 1.01	0.99 ± 0.85	0.95 ± 1.09	1.07 ± 1.09	0.38
BG count, postintervention^∗∗^	0.86 ± 0.9	0.97 ± 0.94	0.78 ± 0.82	0.92 ± 1.06	0.8 ± 0.81	0.48
BG count change	−0.19 ± 0.82	−0.25 ± 0.74	−0.21 ± 0.85	−0.03 ± 0.8	−0.28 ± 0.85	0.26

Mean BG (mg/dL)						
*N*	322	75	112	71	64	
Mean BG, preintervention^∗^	168 ± 50	172 ± 65	170 ± 43	159 ± 40	169 ± 51	0.43
Mean BG, postintervention^∗∗^	158 ± 46	168 ± 51	165 ± 51	148 ± 36	146 ± 40	**0.007**
Mean BG change	−8.3 ± 44	−4.2 ± 52	−2.8 ± 47	−11.3 ± 35	−19.4 ± 35	**0.02**

Estimated HbA1c, eA1c (%)						
*N*	275	75	87	59	54	
eA1c, preintervention	7.5 ± 1.9	7.6 ± 2.1	7.8 ± 1.8	7.2 ± 1.6	7.5 ± 1.8	0.33
eA1c, postintervention	7.1 ± 1.4	7.5 ± 1.3	7.3 ± 1.4	6.9 ± 1.5	6.6 ± 1.3	**0.003**
eA1c, change	−0.4 ± 1.5	−0.1 ± 1.6	−0.4 ± 1.3	−0.4 ± 1.4	−0.7 ± 1.5	**0.02**

All statistically significant comparisons are indicated. ^∗^Average BG checks/day, 30 days prior to intervention. ^∗∗^Average BG checks/day, final 30 days of intervention.

## Supplementary Materials

Supplementary Materials Supplementary Figure 1: raw weight data captured from a single scale. Scale data was cleaned using self-reported weights at registration for reference starting weight; transmitted weight values more than 20% off of the participant's median weight and weight changes greater than 3% over one day were excluded.
