# Supplementary material for: Remote Lifestyle Coaching Plus a Connected Glucose Meter with Certified Diabetes Educator Support Improves Glucose and Weight Loss for People with Type 2 Diabetes
Source: J Diabetes Res. 2018 May 16;2018:3961730. doi: 10.1155/2018/3961730 (PMC5977036; doi:10.1155/2018/3961730)

**Supplementary Figure 1.** Raw weight data captured from a single scale. Scale data was cleaned using self-reported weights at registration for reference starting weight; transmitted weight values more than 20% off of the participant’s median weight and weight changes greater that 3% over one day were excluded.

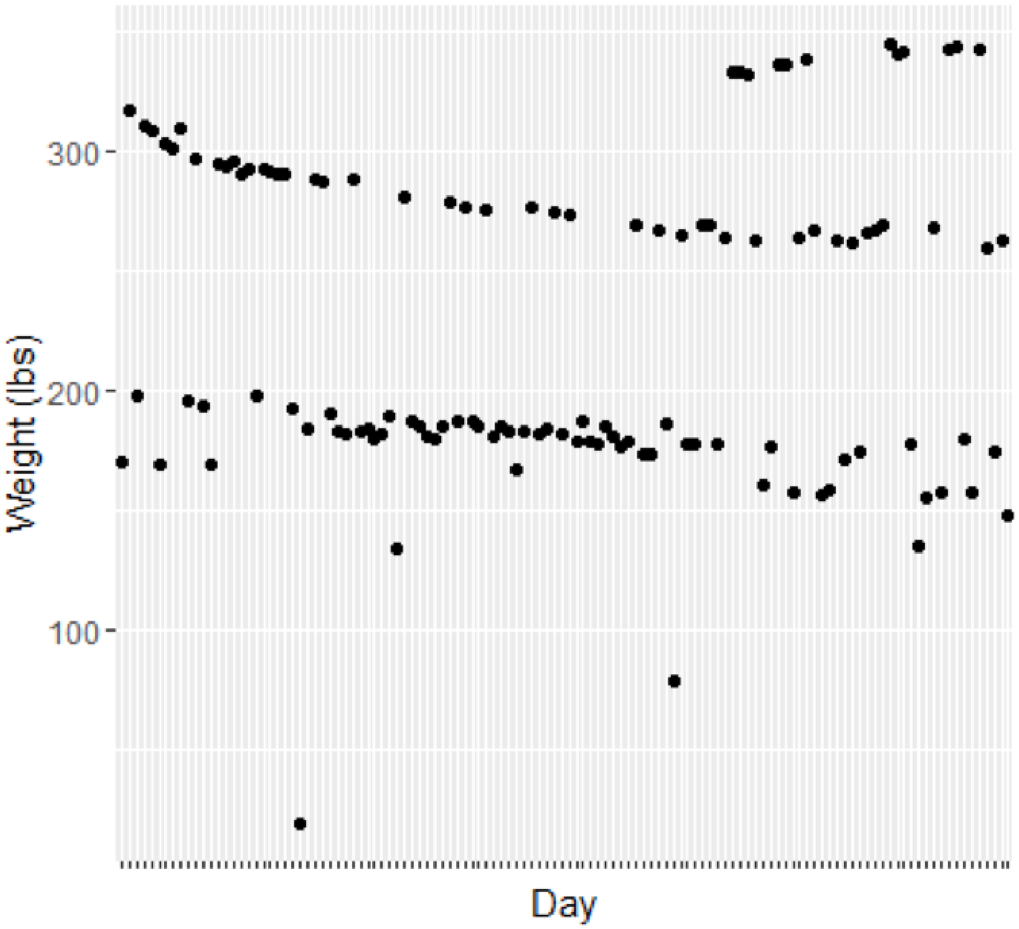

Supplement: Supplementary Materials — Supplementary Figure 1: raw weight data captured from a single scale. Scale data was cleaned using self-reported weights at registration for reference starting weight; transmitted weight values more than 20% off of the participant's median weight and weight changes greater than 3% over one day were excluded. [file 3961730.f1.pdf]
